# Editorial: Design of novel inhibitors for ischemia/reperfusion injury targeting ferroptosis

**DOI:** 10.3389/fphar.2024.1475526

**Published:** 2024-08-21

**Authors:** Xuyang Wang, Qiming Deng, Jingchen Hou, Kalyan Kumar Pasunooti, Kevin Bermea, Kai Li, Genaro A. Ramirez-Correa, Xiaomei Yang, Jianbo Wu

**Affiliations:** ^1^ School of Medicine, Cheeloo College of Medicine, Shandong University, Jinan, Shandong, China; ^2^ Department of Anesthesiology, Shandong Provincial Qian Foshan Hospital, Shandong University, Jinan, Shandong, China; ^3^ Department of Anesthesiology, Qilu Hospital of Shandong University, Jinan, Shandong, China; ^4^ National Key Laboratory for Innovation and Transformation of Luobing Theory, The Key Laboratory of Cardiovascular Remodeling and Function Research, Chinese Ministry of Education, Chinese National Health Commission and Chinese Academy of Medical Sciences, Jinan, China; ^5^ Department of Cardiology, Qilu Hospital of Shandong University, Jinan, China; ^6^ Swarthmore College, Swarthmore College, Swarthmore, PA, United States; ^7^ School of Medicine, Johns Hopkins University, Baltimore, MD, United States; ^8^ Division of Cardiology, The Johns Hopkins University Medical Institutions, Baltimore, MD, United States; ^9^ Division of Human Genetics/Primary and Community Care ISU, School of Medicine, The University of Texas Rio Grande Valley Edinburg, Edinburg, TX, United States; ^10^ Department of Anesthesiology, The First Affiliated Hospital of Shandong First Medical University, Jinan, Shandong, China; ^11^ Shandong Institute of Anesthesia and Respiratory Critical Care Medicine, Jinan, China; ^12^ Shandong Provincial Clinical Research Center for Anesthesiology, Jinan, Shandong, China

**Keywords:** ischemic cardiovascular disease, ferroptosis, ischemia/reperfusion injury, inhibitor, activating transcription factor 3

## Introduction

Ischemic cardiovascular disease, a disease caused by reduced or interrupted blood flow to the heart and other cardiovascular areas, resulting in ischemia of the heart muscle tissue, remains the leading cause of death globally, as it accounts for nearly 50 percent of annual deaths from non-communicable diseases (Sacco et al., 2016). Current prevention and treatment of heart disease include lifestyle changes (Yang et al., 2023), medications, and surgical procedures, which are effective in relieving symptoms and reducing mortality, but also have their limitations and risks. Currently, the mechanisms of cardiovascular disease progression are not well understood, and research is urgently needed. Ferroptosis is an iron-dependent and non-apoptotic form of cell death that has only been defined in recent years and is characterized by the intracellular accumulation of free iron, which leads to the accumulation of reactive oxygen species (ROS) and lipid peroxidation (Dixon et al., 2012), resulting in the rupture of the mitochondrial outer membrane (Mou et al., 2019) and a decrease in the membrane potential (Koppula et al., 2020). In recent years, with the deepening research on ferroptosis, it has been found that ferroptosis plays a crucial role in Ischemic cardiovascular disease and many drugs such as propofol (Li et al., 2022) have been shown in numerous studies to protect the myocardium from ischemia-reperfusion injury by inhibiting ferroptosis. The development of a novel, convenient, and efficient therapeutic approach to treating Ischemic cardiovascular disease by treating ferroptosis has become a hot Research Topic.

Therefore, we organized a Research Topic entitled “Design of Novel Inhibitors for Ischemia/Reperfusion Injury Targeting Ferroptosis”. By collecting articles on applying novel Ferroptosis inhibitors in cardiac ischemia/reperfusion injury, we aim to link cardiac ischemia/reperfusion injury with Ferroptosis. We have collected four high-quality related papers, which are briefly summarized below.

### Pyrroloquinoline quinone and diabetic cardiomyopathy targeting ferroptosis

Zhou et al. revealed that pyrroloquinoline quinone (PQQ) protects the myocardium from hypertrophic damage by modulating Yes-associated Protein (YAP)-associated anti-Ferroptosis activity through an *in vivo* model of transverse aortic constriction (TAC) in mice *versus* an *in vitro* model of phenylephrine (PE)-stimulated neonatal mouse cardiomyocytes. In a previous study (Qu et al., 2022), PQQ has been shown to alleviate diabetic cardiomyopathy (DCM) in diabetic mice by inhibiting the ROS-NF-κB/NLRP3-mediated cellular pyroptosis pathway, suggesting that long-term dietary supplementation with PQQ may be effective in the treatment of DCM. In the present study, PQQ treatment was shown to have a significant effect in inhibiting the accumulation of ferric ions in myocardial tissues after trans-TAC surgery and the increased levels of malondialdehyde (MDA), an anti-oxidative stress marker, while significantly enhancing cellular antioxidant capacity as evidenced by increased glutathione (GSH) levels. In addition, PQQ further enhanced the resistance of cardiomyocytes to Ferroptosis by up-regulating a series of anti-Ferroptosis-related proteins, such as glutathione peroxidase 4 (Gpx4), Ferroptosis suppressor protein 1 (FSP1) and coenzyme Q10 (CoQ10). Meanwhile, PQQ effectively inhibited the activation of YAP in cardiac hypertrophic tissues, showing its protective value in myocardial ischemic disease.

### Activating transcription factor 3 (ATF3) contributes to ferroptosis among sorafenib-induced cardiac injury

Li et al. investigated the role of ATF3 in promoting ferroptosis in sorafenib-induced cardiotoxicity by establishing a mouse model of sorafenib-induced cardiac injury with and without Ferrostatin-1 (Fer-1) pretreatment *in vivo* and *in vitro* and comparing the data from the GEO database (GSE146096). The analysis concluded that ATF3 contributes to the development of Ferroptosis in sorafenib-induced cardiotoxicity by inhibiting the expression of Slc7a11 and that targeted inhibition of ATF3 expression to attenuate ferroptosis provides a new therapeutic strategy for the clinical treatment of sorafenib-induced cardiotoxicity.

### Resveratrol protects myocardial infarction by inhibiting ferroptosis

Liu et al. on the other hand, focused on exploring the role of Resveratrol (Res) in myocardial infarction (MI) in modulating Ferroptosis with myocardial injury and fibrosis. Experiments were conducted using an SD rat myocardial infarction model with ligated left anterior descending branch (LAD) *versus* a cellular Oxygen-Glucose Deprivation (OGD) model with the H9C2 model, it was verified that Res partially reversed the phenotype of myocardial infarction in rats by inhibiting ferroptosis through enhancing KAT5/GPX4 expression. Therefore, this experiment clarified the mechanism of action of Res in MI and provided new targets and pathways for the clinical treatment of MI.

### Allicin improves atherosclerosis by regulating the ferroptosis

Gao et al. employed a combination of network pharmacology, bioinformatics, and experimental validation to comprehensively investigate the active components of garlic, particularly Allicin, in mitigating atherosclerosis (AS) through ferroptosis mediated pathway. Reactive oxygen species (ROS) play a crucial role in AS (Batty et al., 2022), and studies have shown that garlic supplementation can enhance total antioxidant capacity and reduce malondialdehyde (MDA) levels (Moosavian et al., 2020), thereby improving antioxidant effects. Initially, the researchers identified potential AS target genes associated with garlic’s active components from multiple databases. They then used Cytoscape software for network analysis to identify key genes and pathways. Following this, molecular docking techniques and bioinformatics methods were utilized to further evaluate and validate the main active components and their targets. Finally, the therapeutic effects and mechanisms of garlic on AS were assessed through cell and animal experiments. This study reveals the antioxidative and ferroptosis-inhibiting effects of garlic in the prevention and treatment of AS, providing important theoretical support for the development of new targeted drugs against AS.

## Conclusion and perspective

**FIGURE 1:**
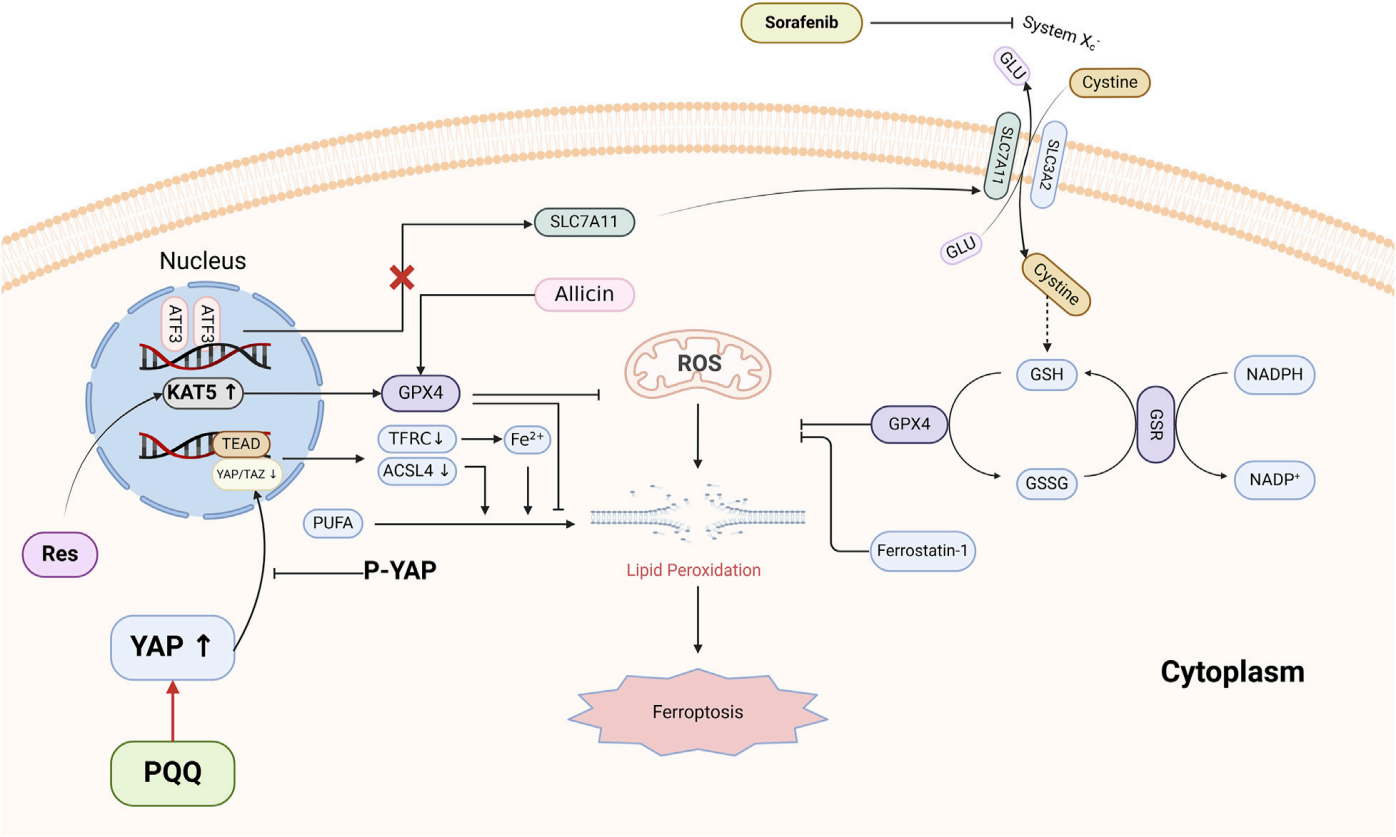
Mechanisms of PQQ, ATF3, Res and Allicin in ferroptosis.

In summary, the articles collected on this provide comprehensive and novel content on the use of Ferroptosis inhibitors in cardiac Ischemia/Reperfusion Injury. The mechanism diagram is summarized in Figure 1. These articles investigated different Ferroptosis pathway target molecules, such as YAP, ATF3, KAT5, etc., and the study of novel ferroptosis inhibitors not only helps to further explore the mechanism and pathway of Ferroptosis in cardiac Ischemia/Reperfusion Injury, but also provides a new approach for the clinical treatment of Ischemic Cardiovascular Diseases by providing new therapeutic strategies and methods. We hope that our research on this topic will bring benefits to patients and the field of ischemic cardiovascular diseases.
